# The psychological conditions for employee engagement in organizational change: Test of a change engagement model

**DOI:** 10.3389/fpsyg.2023.1071924

**Published:** 2023-01-20

**Authors:** Simon L. Albrecht, Sarah Furlong, Michael P. Leiter

**Affiliations:** ^1^School of Psychology, Deakin University, Geelong, VIC, Australia; ^2^Positive Psychology Centers, Melbourne, VIC, Australia

**Keywords:** employee attitudes to change, change engagement, psychological conditions, meaningful work, psychological safety, change self-efficacy, proactive work behavior psychological conditions for employee change engagement

## Abstract

In the contemporary world of work, organizational change is a constant. For change to be successful, employees need to be positive about implementing organizational change. Change engagement reflects the extent to which employees are enthusiastic about change, and willing to actively involve themselves in promoting and supporting ongoing organizational change. Drawing from Kahn’s engagement theory, the research aimed to assess the influence of change-related meaningful work, psychological safety, and self-efficacy as psychological preconditions for change engagement. The study also aimed to test the indirect associations of the change-related psychological preconditions with proactive work behavior through change engagement. Survey data from a Prolific sample (*N* = 297) were analyzed using confirmatory factor analysis and structural equations modeling. In support of the validity of the model, the results showed that change-related self-efficacy, psychological safety, and meaningfulness had significant direct effects on change engagement, explaining 88% of the variance. The change-related psychological conditions also had significant indirect effects on proactive work behavior through change engagement. The findings therefore suggest that employees who exhibit higher levels of change-related self-efficacy, psychological safety, and work meaningfulness are more likely to support and promote organizational change, and to proactively engage in innovative work behavior. In practical terms, organizations that create the psychological conditions for change could significantly improve employee motivation to change and to innovate, which in turn would increase the likelihood of successful organizational change, and improved organizational competitiveness. Study limitations and directions for future research are discussed.

## Introduction

Contemporary organizations operate within the context of constant change (Tsaousis and Vakola, 2018; Kebede and Wang, 2022). In the “new world of work,” organizations have had to quickly adapt to changes wrought by COVID-19, climate change, changes in global markets, global competition, technological advancements, changes to government regulations, and rapidly changing employee expectations and demands (Collins et al., 2020; Malhotra, 2021).

Despite the increasing recognition that change is a constant in contemporary organizational contexts, it has been estimated that change initiatives have a failure rate of between 30 and 70% (Stouten et al., 2018; Schwarz et al., 2021; Hughes, 2022). Although successful organizational change is in large part dependent on how senior leaders envision, plan, implement, and communicate change (Oreg et al., 2011), successful change fundamentally relies on employees adopting new ways of thinking and behaving (Choi, 2011). It is therefore important to draw from relevant theories and research to identify the factors that most influence positive employee attitudes to change (Straatmann et al., 2016). Furthermore, given the importance of employee proactivity and innovation to organizational competitiveness (Anderson et al., 2014), it is also important to identify if positive employee attitudes to change are associated with adaptive performance outcomes such as increased employee proactivity and innovation.

In this paper, we aim to make a number of contributions to the organizational change literature. First, we aim to further establish the construct and a measure of change engagement (Albrecht et al., 2022b). Second, and drawing from the employee change and employee engagement literatures (e.g., Kahn, 1990; Albrecht et al., 2020), we identify three change-related psychological resources (change-related meaningful work, change-related psychological safety, and change-related self-efficacy) that we propose will directly link with and underpin change engagement. Third, we test the validity of brief measures of the three psychological conditions. Fourth, and consistent with engagement theory (Bakker and Demerouti, 2017), we establish whether change engagement is associated with employee proactive work behavior (Griffin et al., 2007) and if change-related psychological resources are indirectly associated with proactive work behavior through change engagement.

In summary, given that organizational change is a constant, and given the importance of positive employee attitudes to successful organizational change, the study aims to fill a gap in the literature by identifying key psychological states that are associated with employee change engagement and proactive work behavior. The key psychological states are drawn from a widely validated theory of work engagement (Kahn, 1990), and adapted to the context of organizational change.

### Employee engagement and psychological resources

Employee engagement is a positive and fulfilling, work-related state of mind whereby employees feel motivated and enthusiastic, and are actively involved in their work (Albrecht, 2010). Beyond energy and active involvement, engagement has also been defined and measured with “absorption” included as a third dimension of engagement (Schaufeli et al., 2002). Absorption refers to a flow-like such that employees feel fully concentrated on, and happily engrossed in, their work. However, engagement researchers have questioned whether absorption is a core aspect of work engagement or an outcome of energy and involvement (Bakker et al., 2011). Meta-analytic research has clearly shown employee engagement to be positively associated with important employee outcomes, such as task performance, contextual performance, proactive work behavior, innovative work behavior, and reduced turnover intentions (e.g., Crawford et al., 2010; Borst et al., 2020; Kwon and Kim, 2020; Neuber et al., 2022; Woznyj et al., 2022).

Much of the academic research focused on identifying the factors that predict engagement has been based in engagement theory (Kahn, 1990), Job Characteristics theory (Hackman and Oldham, 1980), and Job-Demands Resources theory (JD-R; Bakker and Demerouti, 2017). Kahn, for example, proposed psychologically meaningful work, psychological safety, and the availability of physical, emotional, and psychological resources as key enabling conditions for personal engagement at work. May et al. (2004) provided empirical support for Kahn’s propositions. Along similar lines, research has established that personal resources, such as meaningful work, optimism, self-efficacy, and psychological capital also have a direct influence on work engagement (Alessandri et al., 2018; Borst et al., 2020; Mazzetti and Schaufeli, 2022). Psychological conditions and personal resources differ from personality traits in that they are more context specific, labile, and amenable to change (Luthans et al., 2007.)

### Change engagement

Beyond the well-established construct of work engagement, researchers have also argued in support of more “domain-specific” engagement constructs, such as team engagement, engaging leadership, safety engagement, pro-environmental engagement, and change engagement (e.g., Richardson and West, 2010; Schaufeli, 2015; Albrecht et al., 2022a,b). Change engagement has been defined as “an enduring and positive work-related psychological state characterized by a genuine enthusiasm and willingness to support, adopt and promote organizational change” (Albrecht et al., 2020, p. 4). Change engagement consists of two sub-dimensions: change energy and active involvement. The “energy” sub-dimension reflects employee enthusiasm for change, and the “active involvement” sub-dimension reflects an active striving toward the achievement of successful change. The two sub-dimensions are direct analogues of vigor and dedication, the primary sub-dimensions of engagement (Bakker et al., 2011). Change engagement therefore provides a more theoretically grounded and motivational expression of previously researched positive change-related constructs, such as openness to change, readiness for change, and commitment to change (Bouckenooghe et al., 2009; Arnéguy et al., 2018; Tsaousis and Vakola, 2018). Although researchers have shown that change-related organizational and job resources are associated with change engagement (Albrecht et al., 2022a), no research has yet examined the influence of a theoretically coherent set of change-related psychological resources on change engagement.

### Change-related psychological resources and change engagement

**Figure 1:**
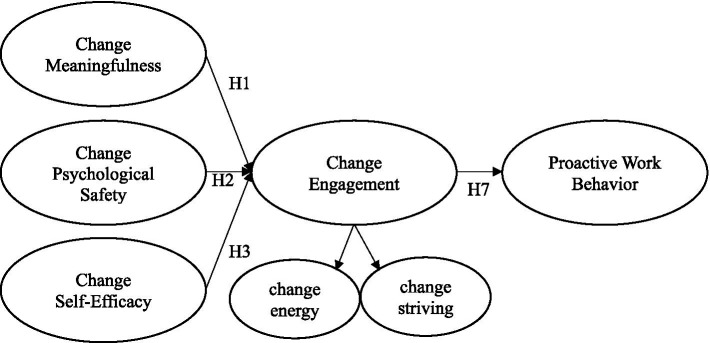
Proposed model. H2, H4, and H6 indirect effects not modeled for ease of representation.

Change-related psychological conditions, or personal resources, refer to “enduring psychological states, or mindsets, that shape an individual’s ability to successfully adapt to a changing work environment” (Albrecht et al., 2020, p. 11). Change-related personal resources include constructs, such as change-related self-efficacy (e.g., Jimmieson et al., 2004), change-related resilience (e.g., Braun et al., 2017), change-related meaning-making (van den Heuvel et al., 2009), and change-related psychological safety (Singh et al., 2018; Albrecht et al., 2020). As domain-specific analogues of psychological conditions for engagement of Kahn (1990), it is here argued that change meaningfulness, change-related psychological safety, and change self-efficacy are core change-related personal resources that are prerequisite conditions for change engagement. Although researchers have suggested that consideration of change-related personal resources is essential to understanding employee attitudes to change (van den Heuvel et al., 2009), researchers have yet to examine the associations between theory-based change-related psychological resources and change engagement, nor their direct or indirect associations with proactive work behavior. Each of these constructs and their proposed relationships are shown in Figure 1 and overviewed below.

#### Change meaningfulness

Meaningful work has been recognized as a fundamental psychological need that underpins individual self-worth and personal agency (Fletcher and Schofield, 2019). Meaningful work has been defined in terms of employees feeling they make an important and useful contribution to a worthwhile purpose though the execution of their work activities (Kahn, 1990; Albrecht et al., 2021). Along similar lines, change meaningfulness reflects the extent to which employees perceive that organizational change will have a positive impact on the contribution they make through their work (Albrecht et al., 2020). As shown in Table 1, change-related meaningfulness is reflected in items such as “changes at work generally mean that I can make a more positive contribution” and “changes at work make the work I do more meaningful for me.”

**Table 1:** Fit indices for alternative measurement and structural models.

Model	χ^2^	df	χ^2^/df	TLI	CFI	RMSEA [90% CI]	SRMR
*Measurement models*							
Proposed	219.341	121	1.813	0.977	0.981	0.052 [0.041, 0.063]	0.0323
Null	5447.029	153	35.601	-	-	0.342 [0.334, 0.350]	-
Single factor	2530.758	135	18.746	0.487	0.547	0.245 [0.237, 0.253]	0.1384
*Structural models*							
Proposed Model	254.205	127	2.002	0.971	0.976	0.058 [0.048, 0.069]	0.0516
Partial Mediation	240.958	124	1.943	0.973	0.978	0.056 [0.046, 0.067]	0.0437

TLI, Tucker-Lewis index; CFI, comparative fit index; RMSEA, root mean square of approximation [90%CI = confidence interval]; SRMR, standardized root mean residual.

Extrapolating from previous research findings (May et al., 2004; van den Heuvel et al., 2009; Schwarz et al., 2021), it is proposed that change meaningfulness will be positively associated with positive attitudes to change in the form of change engagement (H1). In support of the proposition, and drawing from engagement theory of Kahn (1990), May et al. demonstrated a strong association between the psychological condition of meaningfulness and employee engagement. From a more change focused perspective, Schwarz et al. (2021) acknowledged a link between the meaning change recipients assign to change, and their attitudes, cognitions, and emotions about change. Similarly, van den Heuvel et al. (2009) showed that change-related meaning-making is associated with positive employee attitudes toward change. Furthermore, and consistent with Job Characteristics Theory, JD-R theory and the modeling in Figure 1, research has shown that motivational states such as engagement can fully mediate the influence of psychological conditions such as work meaningfulness on performance outcomes such as task performance and extra-role performance (Allan et al., 2019; Kwon and Kim, 2020; van den Heuvel et al., 2020). It is therefore also here proposed that change engagement will fully mediate the relationship between change-related meaningfulness and proactive work behavior (H2).

#### Change-related psychological safety

Change researchers and practitioners have recognized psychological safety as an important component of successful organizational change. Schein and Bennis (1965), in their seminal work on psychological safety and change, defined psychological safety in terms of the extent that individuals feel secure and confident in their ability to manage change. Schein and Bennis argued that psychological safety can remove threats and barriers to change and create a context that “tolerates failure without retaliation, renunciation, or guilt” (p. 45). More broadly, Kahn (1990) defined psychological safety as “feeling able to show and employ one’s true self without fear of negative consequences to self-image, status, or career” (p. 708).

Implicit in the proposed association between psychological safety and employee attitudes to change is the assumption that employees perceive it is psychologically safe for innovating, risk taking and the adoption of change (Kahn, 1990; Frazier et al., 2017). In this sense, change-related psychological safety is a psychological condition for change (Kahn, 1990). Consistent with this proposition, research has demonstrated employee psychological safety to be positively associated with performance and innovation (Baer and Frese, 2003; Frazier et al., 2017). Although May et al. (2004) showed that psychological safety was also positively associated with employee engagement, as yet, no research has been conducted on the construct of change-related psychological safety or its association with positive employee attitudes to change (Albrecht et al., 2022a). As modeled in Figure 1, the current research aims to address the gap by testing the proposed association between change related psychological safety and change engagement (H3). Furthermore, and consistent with job demands-resources theory (Bakker and Demerouti, 2017), it is also proposed that change engagement will fully mediate the relationship between change-related psychological safety and outcome variables such as proactive work behavior (H4).

Consistent with Kahn’s work role centered conceptualization of engagement, May et al. (2004) measured psychological safety using items such as “I am not afraid to be myself at work” and “there is a threatening environment at work (reverse coded).” For present purposes, and consistent with definition of psychological safety of Schein and Bennis (1965), change-related psychological safety is reflected in items such as “I am not afraid to express my opinions about change at work” and “I am confident I would not experience any reprisal or negative consequences if I voiced any concerns about proposed organizational change” (see Table 1). Positive responses to such items, as opposed to reflecting resistance to change, reflect employees feeling comfortable, confident, and willing to participate in change. Employees who participate in change feel more positive about change (Rafferty and Jimmieson, 2018) and are therefore more likely to be positively engaged in change.

#### Change-related self-efficacy

Generalized self-efficacy (Bandura, 1977), as a personal resource, has been shown to be positively associated with work outcomes, such as performance, productivity, and engagement (Xanthopoulou et al., 2007). As a domain-specific sub-set of generalized self-efficacy, change-related self-efficacy has been defined as an employee’s level of self-confidence to effectively deal with change within a changing work environment (Jimmieson et al., 2004; Mehreen et al., 2020).

Change-related self-efficacy, as a construct, to a large extent conceptually parallels Kahn’s psychological condition of availability of resources. May et al. (2004), in their test of engagement model of Kahn (1990), measured availability of resources with items clearly reflecting a “confidence” to deal effectively with changing situations and circumstances: “I am confident in my ability to handle competing demands at work” and “I am confident in my ability to deal with problems that come up at work.” As such, employees with high levels of change-related self-efficacy are more likely to be confident in their ability to manage change effectively (Bandura, 1989; Jimmieson et al., 2004). Given that change-related self-efficacy has been found to positively influence change-related attitudes such as change readiness (Lizar et al., 2015), it is here proposed that change-related self-efficacy will be positively associated with change engagement (H5). Furthermore, and as per JD-R theory (e.g., Xanthopoulou et al., 2007), it is also proposed that change-self efficacy will be indirectly associated with proactive work behavior through its association with change engagement (H6).

### Proactive work behavior

Griffin et al. (2007) distinguished between three forms of work performance: proficiency (task performance), adaptivity (responses to accommodate change), and proactivity (self-initiated change). Proactive performance manifests as change-oriented behaviors that reflect an employee’s willingness to initiate and adopt new and innovative ways of thinking and behaving that can lead to organizational performance and competitive advantage (Straatmann et al., 2016; Straatman et al., 2018). Griffin et al. (2007) measured proactive behavior with items assessing how often respondents have “come up with ideas to improve the way in which your core tasks are done” and “made suggestions to improve the overall effectiveness of the organization (e.g., by suggesting changes to administrative procedures).” Given that researchers have argued and demonstrated that employee engagement leads to innovative and proactive work behaviors (Hakanen et al., 2008; Salanova and Schaufeli, 2008), it is here proposed that change engagement will be positively associated with proactive work behavior (H7). Furthermore, and as previously noted as H2, H4, and H6, theory and research suggest that the influence of psychological conditions, such as meaningfulness, psychological safety, and self-efficacy will influence outcomes such as proactive behavior through their influence on engagement (Allan et al., 2019; Kwon and Kim, 2020; van den Heuvel et al., 2020).

Overall, the present study aimed to address gaps in the literature by extending well established engagement theories to the context of organizational change. The research tests a model proposing positive associations between change-related psychological resources, positive attitudes to change, and employee performance. More specifically, it is proposed that positive employee experiences of change-related meaningful work, psychological safety, and self-efficacy, will be directly associated with employee change engagement, and indirectly associated with self-reported proactive work behavior (see Figure 1).

## Methods

### Participants and procedures

The sample consisted of 301 paid participants sourced from Prolific. Research has shown that Prolific data have “similar psychometric properties and produces criterion validities that generally fall within the credibility intervals of existing meta-analytic results from conventionally sourced data” (Walter et al., 2019, p. 425). Beyond having a good record of responding to survey questions, the participants needed to be 18 years or older, and working full- or part-time for a minimum of 3 months in an organization of 15 or more employees that had experienced organizational change within the past year. Prior to responding, participants were informed about the purpose of the study, the confidentiality of their responses, and that the study had been granted ethics approval. Participants were also informed before commencing the survey that they needed to attend carefully to, and complete, all survey questions to receive payment. Of the 297 participants who satisfied the inclusion criteria, 49.2% reported as female and 49.8% as male. Ages ranged from 18 to 66 years, with a mean of 36.5 years. Tenure ranged from 6 months to 40 years, with a mean of 6 years. Respondents worked in organizations of varying size (10–1 million plus employees) as team members (53%), senior managers (5%), managers (17.5%), team leaders (17.8%), and executives (2%). The clear majority of participants (79%) reported experiencing a moderate to a great deal of organizational change during the previous 12 months (e.g., new structures, restructures, mergers, new technologies, new systems, new policies, relocations, changes to work schedules, and leadership).

Online calculator of Soper (2022) showed that the sample size used in the analyses (*N* = 297) exceeded the minimum sample size (*N* = 200) needed to test the proposed model at power and to detect a medium effect size. Previous studies exploring the influence of psychological conditions on engagement and change have reported medium to large effect sizes (e.g., May et al., 2004; van den Heuvel and Demerouti, 2009).

### Measures

The six first order constructs shown in Figure 1 were measured on a seven-point Likert scale (1 = strongly disagree to 7 = strongly agree). Items included in each of the scales and their factor loadings are shown in Table 1. Scale means, standard deviations, alpha reliabilities, and correlations are shown in Table 2.

**Table 2:** CFA items and standardized loadings.

Scale	Item	Loadings
*Change-related psychological conditions*		
Meaning 1	Changes at work make the work I do more meaningful for me.	0.883
Meaning 2	Changes at work generally mean that I can make a more positive contribution.	0.952
Meaning 3	Changes at work usually result in job having a more positive impact on clients or customers inside or outside the organization.	0.847
Safety 1	I am not afraid to express my opinions about change at work.	0.925
Safety 2	I am confident I would not experience any reprisal or negative consequences if I voiced any concerns about proposed organizational change.	0.958
Safety 3	I feel safe being open and frank about my opinions about organizational changes.	0.906
Self-Eff 1	I am able to successfully overcome any challenges associated with organizational change.	0.903
Self-Eff 2	I am confident in my ability to implement any change initiatives that this organization promotes.	0.852
Self-Eff3	When we are going through organizational change, I feel confident I can work through problems to find solutions.	0.931
*Change Engagement—Energy*		
	I am enthusiastic about change in this organization.	0.907
	I feel energized when we are going through change.	0.895
	I feel positive about changes when they occur in this organization.	0.932
*Change Engagement—Active Involvement/Striving*		
	I strive as hard as I can to contribute positively to change initiatives in this organization.	0.854
	I actively involve myself in changes that take place in this organization.	0.846
	I strive to make sure change is implemented successfully in this organization.	0.925
*Proactive Work Behavior*		
	I initiated better ways of doing my main work tasks.	0.875
	I came up with ideas to improve the way in which my work is done.	0.941
	I made changes to improve the way my main work tasks are done.	0.929

Meaning, change meaningfulness; Safety, change-related psychological safety; and Self-Eff, change-related self-efficacy.

#### Change-related psychological resources

Change-related self-efficacy, change-related meaning, and change-related psychological safety were measured with three-item scales adapted from May et al. (2004). May et al. reported Cronbach alpha coefficients ranging from 0.71 to 0.90 for their corresponding measures of the psychological conditions of engagement.

#### Change engagement

Change engagement was measured with two three-item sub-scales (Albrecht et al., 2022a), with the items adapted from measures of job engagement (Rich et al., 2010) and employee attitudes to change (e.g., Bouckenooghe et al., 2009; Tsaousis and Vakola, 2018). As shown in Table 1, items include “I am enthusiastic about change in this organization” and “I strive as hard as I can to contribute positively to change initiatives in this organization.” Albrecht et al. reported alpha reliabilities across two samples ranging between 0.90 and 0.93 for the two sub-scales. The higher order modeling of change engagement (see Figure 1) is consistent with engagement research showing that engagement, as a higher order construct, explains the covariation between first order constructs of vigor, dedication, and absorption (e.g., Schaufeli et al., 2006).

#### Proactive work behavior

Proactive work behavior was measured with three items adapted from Griffin et al. (2007). Example items included “I initiated better ways of doing my main work tasks” and “I came up with ideas to improve the way in which my work is done.” Griffin et al. (2007) reported an alpha of 0.90 for their similar three-item measure.

### Data analytic strategy

The two-step modeling approach of Anderson and Gerbing (1988) was applied to test the theoretically based model. As a first step, Confirmatory Factor Analysis (CFA) was used to assess the fit of the measurement model. Given the cross-sectional correlational survey design, the CFA analytic strategy also included assessing the influence of common method bias (Podsakoff et al., 2012). As a second step, structural equation modeling (SEM) was conducted to test the proposed relationships shown in Figure 1. CFA and SEM analyses were conducted using conventional or covariance based structural equation (CB-SEM) with AMOS v26 and Maximum Likelihood estimation.

Both the CFA and SEM models were assessed using a number of recommended fit indices: the chi-square statistic (non-significant), chi-square to degrees of freedom ratio (< 2 or 3; Jaccard et al., 1996), Tucker Lewis Index (TLI > 0.95), Comparative Fit Index (CFI > 0.95), the root mean square error of approximation (RMSEA < 0.05; Hu and Bentler, 1999), and the standardized root mean residual (SRMR < 0.06; Kline, 2016). Beyond assessing the overall fit and the direct effects within the proposed model, bootstrapping procedures were used to assess the indirect effect of the three change-related psychological resources on proactive work behavior *via* change engagement.

## Results

### Confirmatory factor analysis

As shown in Table 1, the proposed measurement model yielded acceptable fit. The chi-square ratio, TLI, CFI, and SRMR were at, or better than, criterion values. Although the RMSEA estimate was slightly higher than the recommended criterion, a model can be accepted with confidence when a range of different fit indices are at criterion (Hu and Bentler, 1999; Heene et al., 2011). Table 1 also shows that all fit indices were superior to the null model and a one factor model, calculated for comparison purposes. Additionally, and in support of convergent validity, the standardized loadings for all items were strong and statistically significant (*p* > 0.001), ranging from 0.846 to 0.958 (see Table 2).

Table 3 shows the means, standard deviations, Cronbach’s α, average variance extracted, and correlations for all first-order variables included in the measurement model. The α reliabilities exceeded the accepted criterion value of 0.80 (Nunnally, 1978). In support of the convergent validity of the scales, all six AVE values (ranging from 0.77 to 0.86) clearly exceeded the accepted criterion value of 0.5 (Fornell and Larcker, 1981). Furthermore, as shown in Table 3, the bivariate correlations were all significant and positive. Given the relatively strong correlation between change meaningfulness and the energy sub-scale of change engagement (*r* = 0.82), the discriminant validity for these two constructs was tested. That is, the chi-square values were compared when the correlation between the two constructs was first fixed at one, as opposed to when it was freely estimated (Anderson and Gerbing, 1988). The sizable and significant difference between the chi-square values, given one degree of freedom, supported the discriminant validity of the two constructs (Δχ^2^ = 203.7; *p* < 0.001). More broadly, and also in support of discriminant validity, the square root of each construct’s AVE (ranging from 0.88 to 0.93) exceeded all corresponding construct correlations (Fornell and Larcker, 1981).

**Table 3:** Means, SD, Cronbach’s alpha (α), average variance extracted (AVE), and correlations among first-order variables (*N* = 297).

Variable	Mean	SD	α	AVE	1	2	3	4	5	6
1. Change Meaningfulness	4.26	1.54	0.92	0.80	*-*					
2. Δ Psychological Safety	4.44	1.80	0.90	0.86	0.56	-				
3. Δ Self-Efficacy	5.51	1.15	0.92	0.80	0.50	0.44	-			
4. Δ Engagement—Energy	4.37	1.55	0.94	0.83	0.82	0.64	0.61	-		
5. Δ Engagement—Active Involvement/Striving	5.02	1.40	0.90	0.77	0.63	0.56	0.59	0.71	-	
6. Proactive Work Behavior	5.06	1.45	0.94	0.84	0.25	0.27	0.34	0.32	0.44	-

All correlations significant at *p* ≤ 0.001; Δ = Change.

Given the cross-sectional and self-report nature of the data, tests for common method variance (CMV) were conducted by comparing CFA standardized loadings before and after the addition of a latent common method factor (Podsakoff et al., 2012). Relative to the proposed measurement model, the standardized loadings for seven of the 18 items decreased more than 0.20 (range 0.23–0.45) after including the common method factor in the model. The seven items, not surprisingly, included five change engagement items that formed part of the higher order change engagement factor (see Figure 1). The remaining two items were from the change meaningfulness scale, and therefore suggest some potential redundancy between the items. Overall, the standardized loadings decreased by a relatively modest 0.22 on average, and all factor loadings remained statistically significant (*p* < 0.001) after the inclusion of the common method factor. Therefore, although acknowledging the presence of CMV, the method effects appear to not be overly influential (Johnson et al., 2011; Podsakoff et al., 2012). Furthermore, and in support of modeling change-engagement as a higher order construct, the sub-scale loadings on the higher-order factor clearly exceeded the recommended value of 0.70 (Credé and Harms, 2015).

### Structured equation modelling

**Figure 2:**
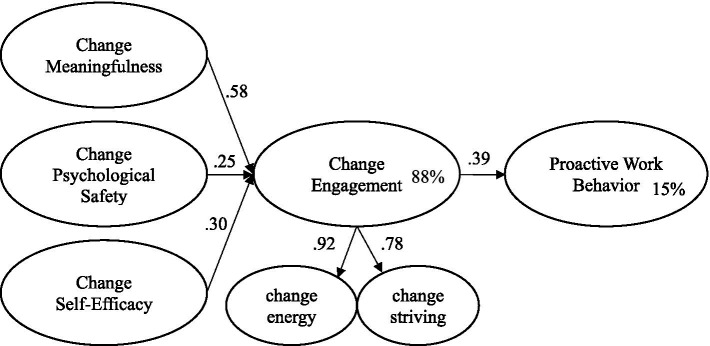
Standardized parameter estimates; percent variance explained. Items and errors not shown for ease of representation.

Having established the validity of the measurement model, step 2 of the broad analytic strategy (Anderson and Gerbing, 1988) involved using SEM to test the fit of the proposed structural model (see Figure 1). The structural model yielded good fit (see Table 1). In support of H1, H3, and H5, Figure 2 shows that as proposed, each of the three psychological conditions (change-related meaning, change-related psychological safety, and change-related self-efficacy) had a significant, positive, and direct effect on change-engagement. In support of H7, change-engagement had a significant, positive, and direct effect on proactive work behavior. The model explained 88.1% of the variance in change engagement and 15% of the variance in proactive work behavior.

To test the proposed mediating influence of change engagement on the relationship between the three change-related psychological resources and proactive work behavior, bias corrected bootstrapping procedures were conducted. In support of H2, H4, and H6, using 2,000 bootstrap samples, significant and positive indirect effects were found for each of the change-related psychological resources: change-related meaning (*β* = 0.224, *p* < 0.001, CI 95% = 0.146–0.312), change-related psychological safety (*β* = 0.096, *p* < 0.001, CI 95% = 0.051–0.164), and change-related self-efficacy (*β* = 0.115, *p* < 0.01, CI 95% = 0.063–0.195). As a final step in the analyses, an alternative partial mediation model was tested whereby all three psychological conditions were also specified to be directly associated with proactive work behavior. Although the partial mediation model provided equally good fit to the data (see Table 1), none of the three additional parameters were significant (*p* = 0.088, 0.272, 0.592). Therefore, the proposed fully mediated model was accepted as the most parsimonious and theoretically defensible model.

## Discussion

The study aimed to test a change focused analogue of engagement theory (Kahn, 1990) whereby three change-related psychological conditions were proposed to directly influence employee change engagement, and to indirectly influence employee performance in the form of proactive work behavior. The results supported the proposed model in that change-related meaning, change-related psychological safety, and change-related self-efficacy each had a direct positive effect on change engagement (H1, H3, and H5). In support of the validity of the model, the change-related psychological resources explained a very substantial 88% of the variance in change engagement. In line with H7, the results also showed that change engagement had a significant positive effect on proactive work behavior (Griffin et al., 2007), a performance outcome widely considered to be important for sustained competitive performance within the contemporary world of work (Carbonell et al., 2016). Furthermore, and in line with H2, H4, and H6, change engagement was shown to fully mediate the associations the three psychological conditions and proactive work behavior. Overall, the results are consistent with well-established theories, such as engagement theory (Kahn, 1990), Job Demands-Resources theory (Bakker and Demerouti, 2017), and Job Characteristics Theory (Hackman and Oldham, 1980) that explain how motivational constructs serve as explanatory mechanisms by which psychosocial job and psychological resources result in performance outcomes. For the present case, employee change engagement, as a motivational construct, served as an explanatory mechanism by which three psychological conditions influenced employee proactive behavior.

Overall, the research makes a number of contributions to the engagement and the organizational change literatures. Given that the engagement and change literatures have largely run along parallel paths (Albrecht, 2021), the research results support the extrapolation of engagement theory to the context of organizational change. Just as engagement theory has established the antecedents and outcomes associated with employees being enthusiastic and actively involved in their work roles, it is equally important to establish the antecedents and outcomes associated with employees feeling enthusiastic and actively involved in ongoing organizational change. Although previous research has included consideration of change-related organizational and job resources (Albrecht et al., 2022a), the current study is the first to assess psychological conditions of Kahn (1990) for work engagement within the context of organizational change.

The results showed that change-related meaningfulness explained considerably more variance in change engagement than did change-related self-efficacy or change-related psychological safety. This finding is consistent with previous research that has highlighted the important contribution that meaningful work activities have on employee engagement, motivation, and performance (May et al., 2004; Allan et al., 2019; Albrecht et al., 2021). The findings therefore extend the change literature by identifying that change initiatives need to result in employees feeling that their work is meaningful, that they can make a meaningful contribution, and that they can have a positive impact on their clients or customers. Employees who experience a heightened sense of purpose and contribution through their experience of organizational change will likely be more willing to engage enthusiastically in organizational change (van den Heuvel and Demerouti, 2009).

The results showed, although to a lesser extent than change meaningfulness, that employees who experience increased change self-efficacy will likely have greater self-confidence in their ability to deal effectively with the situational demands experienced during organizational change (Hicks and Knies, 2015), and will therefore likely be more willing to engage enthusiastically in organizational change. That is, employees who feel able to successfully overcome any challenges associated with organizational change, who are confident in their ability to implement change initiatives, and who feel confident they can successfully work through problems when going through change, are more likely to engage positively in change and perform more proactively in their work.

The finding of a positive association between change-related psychological safety and change engagement is consistent with previous research showing a positive association between psychological safety and employee engagement (e.g., May et al., 2004). The present findings suggest that employees who feel comfortable, confident, and willing to participate in change are therefore more likely to be positively engaged in change. Additionally, and beyond more generic conceptualizations and measures of psychological safety, the current research suggests that domain specific measures of change-related psychological safety influence change-engagement and proactive performance. That is, employees who are not afraid to express their thoughts and opinions about change, and who are confident they would not experience negative consequences if they voiced concerns about proposed organizational change, are more likely to be enthusiastic about change and proactive in their work.

Beyond the direct effects on change engagement, each of the three change-related psychological conditions also had a significant indirect effect on employee proactive work behavior. The results therefore suggest that in order to have employees who are more likely to initiate improved ways of doing their work, it is advisable to ensure employees have the personal resources to contribute meaningfully, safely, confidently, and competently to change (van den Heuvel et al., 2009). As with the direct effects on change engagement, meaningful change had the strongest indirect effect on proactive work behavior.

The present results extend the organizational change literature by focusing on the context of ongoing change. In contrast to previous change literature that has largely focused on employee attitudes to a specific change, respondents were asked to think back over the changes that had occurred over the past 12 months. Some 80 % of respondents reported experiencing a moderate to a great deal of organizational change in the form of restructures, mergers, new technologies, new systems, new policies, relocations, changes to work schedules, and changes in leadership. The model and the measures presented in the current research can therefore be applied more broadly within the contemporary context of constant and ongoing organizational change, as opposed to being restricted to a specific and discrete change event. The research identified that change meaningfulness, change self-efficacy, and change psychological safety are positively linked to employees feeling energized about ongoing organizational change and willing to strive toward the successful achievement of ongoing organizational change.

Beyond identifying three psychological conditions that might help explain the emergence and maintenance of employee engagement in change, the research also makes a contribution to the change literature through the development of measures of change meaningfulness, change self-efficacy, and change psychological safety. Given that confirmatory factor analysis supported the psychometric properties of the measures, researchers, and practitioners will be able to use such measures with a degree of confidence. The CFA and reliability analyses also further corroborated the previously reported validity and reliability of the measure of change engagement (Albrecht et al., 2022a).

### Practical implications

The findings add to the body of research supporting change engagement as a potentially useful resource that organizations can use to succeed in an environment of ongoing change. It has been well established that if employees are ready, open, and committed to change, the chance of change initiatives being successfully implemented is increased (Choi, 2011). Given the very significant investment of financial, technical, and people resources that organizational change demands, it is incumbent on organizations to optimize the return on investments through the successful realization of change. The results of the current study suggest that organizations should build interventions into change programs that are focused on developing employee change meaningfulness, change self-efficacy, and change-related psychological safety. Facilitating employee experiences of change meaningfulness, change self-efficacy, and change-related psychological safety, and particularly meaningful change, will increase the likelihood that employees will be enthusiastic about change, and actively involved in change.

Meta-analytic evidence has shown that both top-down and bottom-up, resource-developing interventions are effective in developing work engagement (Björk et al., 2021). Similar intervention strategies are likely to be effective in developing change-engagement. As such, training, coaching, job crafting, and team-based and organizational learning development interventions that have previously been used to help employees develop work engagement, self-efficacy, work meaningfulness, and psychological safety could usefully be adapted to the context of change. For example, job crafting interventions have been linked to increased employee meaningfulness of work, increased work engagement, and increased PsyCap (Sakuraya et al., 2016; Bruning and Campion, 2019). Bottom-up “change crafting” interventions, paralleling job crafting intervention designs, might involve participants identifying their own realistic and meaningful goals, breaking down key goals into manageable sub-goals, identifying and evaluating multiple pathways to achieve goals and sub-goals, identifying resources needed to achieve the goals, and the sharing of participant experiences (Luthans et al., 2006). Such intervention designs might be applied to help employees enhance change-related job resources, such as change information, change involvement, supervisor support for change, and co-worker support for change (Albrecht et al., 2020; Björk et al., 2021).

In organizational development terms, the research provides brief and reliable measures of change related personal resources and employee change engagement that can be used as “pulse-check” diagnostics of employee attitudes to change (Jolton and Klein, 2020). That is, in addition to assessing the influence of change-related job resources on employee enthusiasm for and involvement in change, survey diagnostics can be administered to assess the extent to which employees are confidently experiencing on-going organizational change as meaningful and psychologically safe. Organizations could then share survey results with employees and involve employees in developing action plans to build on strengths and address threats to employee wellbeing and performance throughout organizational change initiatives.

### Limitations and future research

Despite the contributions of the study, several limitations should be noted. Firstly, the use of the prolific sample may suggest the results may not generalize to the wider world of work. However, as previously noted, researchers have shown that Prolific samples yield results with similar psychometrics and validities to conventionally sourced data (Walter et al., 2019). Secondly, because the data were single-source, and self-report, common method variance may have impacted the validity of the model (Podsakoff et al., 2012). However, as previously noted in the results, tests for common method variance showed that after the inclusion of a common method factor, the CFA standardized loadings decreased by a relatively modest amount, and all factor loadings remained statistically significant. Thirdly, because the data were collected at one time point, no strong claims for causal relations between the constructs can be made. Further research using longitudinal designs would be required to confidently establish the direct and indirect effects reported. Further research could also be conducted to integrate the current research findings within previously proposed research models that have examined the influence of organizational and change-related job resources on change engagement (Albrecht et al., 2022a). Beyond the influence of the three psychological conditions examined in the current study, future research could also examine the mediating influence of additional change-related personal resources such as change-related hope, optimism, and resilience on change engagement and proactive behavior (Youssef and Luthans, 2007). Importantly, further intervention research is needed to establish the effectiveness of top-down and bottom-up interventions such as change crafting as a means to enhance employee change engagement.

### Conclusion

The study aimed to assess the validity of a domain-specific analogue of engagement theory (Kahn, 1990). Consistent with the theory, the results showed that change-related meaningful work, psychological safety, and self-efficacy potentially serve as important psychological preconditions for change engagement and proactive work behavior. Therefore, organizations can expect to significantly improve employee motivation to change and to innovate when they create the conditions whereby employees experience organizational change to have purpose, and whereby employees feel competent, confident, and psychologically safe. Where employees are enthusiastic and motivated about change, there will therefore be an increased likelihood of the successful implementation of organizational change, and therefore improved organizational competitiveness and sustainability.

## Data availability statement

The raw data supporting the conclusions of this article will be made available by the authors, without undue reservation.

## Ethics statement

The study was approved on 08/07/2021 by the Ethics Committee of Deakin University (HEAG-H 60_2021) and adheres to the Australian Codes for the Responsible and Ethical Conduct of Research. The ethics approval is therefore consistent with terms laid out in the Declaration of Helsinki (DoH). The patients/participants provided their written informed consent to participate in this study.

## Author contributions

SA initiated the conceptualization of the project, collected data, conducted analyses, and was responsible for the structure and content of the final manuscript. SF contributed to the structure and content of the final manuscript. ML reviewed the structure and content of the final manuscript and provided support for the publication process. All authors contributed to the article and approved the submitted version.

## Conflict of interest

The authors declare that the research was conducted in the absence of any commercial or financial relationships that could be construed as a potential conflict of interest.

## Publisher’s note

All claims expressed in this article are solely those of the authors and do not necessarily represent those of their affiliated organizations, or those of the publisher, the editors and the reviewers. Any product that may be evaluated in this article, or claim that may be made by its manufacturer, is not guaranteed or endorsed by the publisher.
